# Assessment of Safety, Efficacy, and Functional Outcomes After Rotator Cuff Repair Using Ceptre® Titanium Screw Anchor: A Retrospective Study

**DOI:** 10.7759/cureus.38121

**Published:** 2023-04-25

**Authors:** Karnav A Panchal, Ashok K Moharana, Sachin Angrish, Deepak TS

**Affiliations:** 1 Arthroscopy & Sports Medicine, Epic Hospital, Ahmedabad, IND; 2 Clinical Affairs, Healthium Medtech Limited, Bengaluru, IND

**Keywords:** orthopedic surgical repair, spadi score, ases score, titanium screw anchor, rotator cuff injury

## Abstract

Background

Rotator cuff tears (RCTs) are the most common cause of shoulder disability. RCT is characterized by progressive wear and tear of the tendon tissue over time. The incidence of cuff tears ranges from 5% to 39%. With increasing advancements in the surgical sector, an upward trend has been observed in repair surgeries where torn tendons are repaired arthroscopically by inserting surgical implants. With this background, this study aimed to assess the safety, efficacy, and functional outcomes after RCT repair using Ceptre® titanium screw anchor implants.

Methodology

This retrospective, observational, single-center, clinical study was conducted at Epic Hospital in Gujarat, India. Patients who underwent rotator cuff repair surgery between January 2019 and July 2022 were recruited and followed up in December 2022. Baseline characteristics and surgical and post-surgical details were collected from patient medical reports and post-surgery progress data were documented through telephonic follow-up. The functional outcomes and efficacy of the implant were assessed using the American Shoulder and Elbow Surgeons (ASES) form, Shoulder Pain and Disability Index (SPADI) score, Simple Shoulder Test (SST), and Single Assessment Numeric Evaluation (SANE) score.

Results

The mean age of the recruited patients was 59.74 ± 8.91 years. Among the recruited patients, 64% were females and 36% were males. About 85% of patients had a right shoulder injury and 15% of patients (n = 6/39) had a left shoulder injury. Further, 64% (n = 25/39) of patients had supraspinatus tears, whereas 36% (n = 14) of patients had both supraspinatus and infraspinatus tears. The mean ASES, SPADI, SST, and SANE scores were observed to be 81.43 ± 14.20, 29.41 ± 12.6, 75.41 ± 12.96, and 94.67 ± 7.50, respectively. No adverse events, re-injuries, or re-surgeries were reported by any of the patients during the study period.

Conclusions

Our findings suggest that arthroscopic rotator cuff repair using Ceptre Knotted Ultra-High-Molecular-Weight Polyethylene Suture Titanium Screw Anchor resulted in favorable functional outcomes. Thus, it could be a considerable implant for a successful surgery.

## Introduction

Rotator cuff injuries such as rotator cuff tears (RCTs) are the most common cause of shoulder disability and are a frequently encountered problem in daily orthopedics practice [1]. They are often caused by progressive wear and tear of the tendon tissue over time. The incidence of cuff tears ranges from 5% to 39% [2]. They can be symptomatic or asymptomatic. In a study, Mall et al. (2010) reported that symptomatic tears are bigger in size compared with asymptomatic tears [3]. Due to the asymptomatic nature of the disease, it is difficult to estimate the prevalence of RCTs accurately [4].

Further, the risk of RCT also increases with age [2]. Studies have reported an age-wise prevalence of 9.7% in patients younger than or 20 years of age, 19% in 21-49 years of age, 40.7% in 50-70 years of age, and 62% in patients aged 80 years or older [2,5,6]. RCT is more common in older individuals and those who frequently perform overhead motions, such as painters and carpenters. In a pilot study, Loew et al. analyzed tendon damage in the painter population working for more than 10 years. The authors found supraspinatus tear in 45% of painters compared to 8% in the control group [7]. Repetitive overhead activities, falls, accidents, or sports activities such as baseball, tennis, and weight-lifting can damage the tendon [8].

RCTs are of two types, namely, partial-thickness and full-thickness. Partial-thickness and full-thickness are also known as incomplete and complete tears, respectively. The tendon is attached to the arm bone in a partial tear, whereas the tendon is completely separated from the bone in a full-thickness tear, resulting in a hole or rip in the tendon [3,9]. Partial-thickness tears are more frequent than full-thickness tears, with a prevalence of 13% versus 7%. Small tears may only limit the function but large tears result in an imbalance in the joint kinematics. RCT causes pain and reduces the ability to perform activities of daily living [10,11]. Thus, surgical repair of RCTs is a considerable procedure to alleviate shoulder pain, to rectify tears, and when other treatments fail [9].

In recent decades, there has been an increase in the incidence of patients undergoing surgery with an upward trend [12]. Torn tendons are repaired arthroscopically by inserting surgical instruments and implants, such as metal (titanium or stainless steel), plastic, or bioabsorbable (natural, synthetic, or biosynthetic polymers) suture anchors. These sutures have advantages and disadvantages, for instance, metallic suture anchors provide rigid fixation and release metal ions in their surroundings and may cause difficulty in revision surgery. Conversely, biodegradable sutures have improved biocompatibility and may lead to less complicated revision surgery but may lose their strength shortly after surgery. However, metallic suture anchors have been used successfully for a long time [13]. The long-term clinical results of both arthroscopic and open rotator cuff repairs are good, with more than 90% of good or excellent results at 10 years [13-15].

Furthermore, failed repair and re-tear following RCT repair are major concerns for orthopedic surgeons. Re-tear rate after RCT repair has been reported to range up to 40% for small-to-medium tears and up to 94% for large and chronic tears [16-18]. Despite a large amount of literature on the management of RCTs and with the advancements in the technologies for RCT repair, surgical indications are not standardized.

To address these challenges, Sironix has developed a shoulder implant called Ceptre® Knotted Ultra-High-Molecular-Weight Polyethylene (UHMWPE) Suture Titanium Screw Anchor, which is designed for soft tissue fixation. We conducted a retrospective, observational, single-center study to evaluate the safety, efficacy, and functional outcomes of RCT repair using Ceptre titanium screw anchor implants in patients at Epic Hospital in Gujarat, India.

## Materials and methods

Study design

This retrospective, observational, single-center, clinical study was conducted at Epic Hospital in Gujarat, India among patients who underwent rotator cuff repair surgery between January 2019 and July 2022 and who were followed up until December 2022.

Ethical approval

The study protocol was approved by the Institutional Ethical Committee (Epic Hospital). Before enrolment, verbal consent was obtained during telephonic follow-up. The study was conducted according to the current version of the Declaration of Helsinki and in compliance with the current Indian Council of Medical Research Guidelines for Biomedical Research on Human Patients, and in accordance with Medical Device Directive (MEDDEV) 2.12/2 rev.2 and ISO 14155:2020 (Clinical Investigation of Medical Devices for Human Subjects - Good Clinical Practice).

Selection of study patients

A total of 39 patients who met the following eligibility criteria were enrolled in the study.

Inclusion Criteria

Patients aged ≥18 years who had undergone rotator cuff repair surgery with Ceptre® titanium screw anchor implant between January 2019 and July 2022 were included. Moreover, patients willing to provide verbal consent during a telephonic follow-up were included in this study.

Exclusion Criteria

We excluded patients who had suffered a traumatic injury to the same shoulder after surgery and who could not be contacted after three attempts.

Ceptre® Suture Titanium Screw Anchor

**Figure 1:**
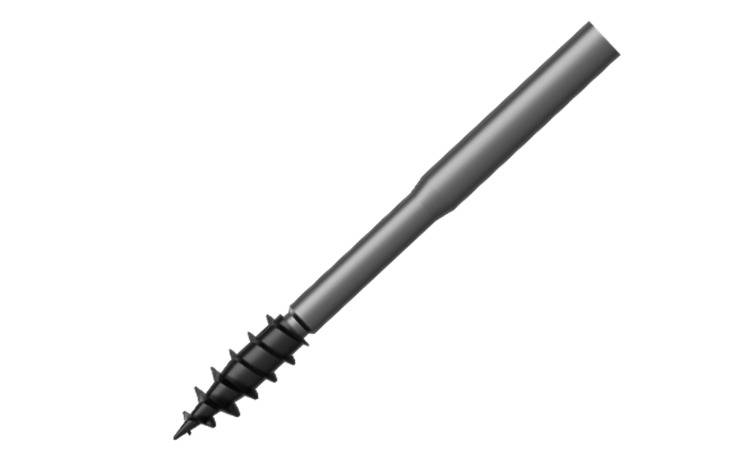
Ceptre® Titanium Screw Anchor implant. Sinorix Arthroscopy Solutions, Healthium Medtech Limited, India.

The Ceptre® Knotted UHMWPE Suture Titanium Screw Anchor (Sinorix Arthroscopy Solutions, Healthium Medtech Limited, India) is a combination of a non-absorbable suture with a knotted anchor made of titanium and preloaded on a disposable inserter assembly intended for fixation of the suture/tape to the bone (Figure 1).

Objectives

In this study, we aimed to evaluate the functional outcomes after rotator cuff repair with Ceptre® Titanium Screw Anchor using the American Shoulder and Elbow Surgeons (ASES) form. We aimed to review the safety of the shoulder implant after rotator cuff repair by evaluating adverse events. Finally, we aimed to review the efficacy of the Ceptre® Titanium Screw Anchor after rotator cuff repair by evaluating the pain and activity level in patients using Shoulder Pain and Disability Index (SPADI) score, Simple Shoulder Test (SST), and Single Assessment Numeric Evaluation (SANE) score.

Data collection

Retrospective data were collected from patients’ medical records and discharge summaries electronically. Baseline parameters such as age, gender, employment status, marital status, surgical procedures, implants used, number of implants, adverse events, and re-tear were documented in a case report form (CRF). Further, the study patients were followed up telephonically to collect the following data using a pre-prepared questionnaire in the form of CRF.

ASES Score

The ASES instrument comprises two sections covering participant self-reported measures and clinician assessments such as participant’s shoulder pain rating (Visual analog scale (VAS) pain) and self-reported ability to perform 10 different activities of daily living (ADLs). The ASES score ranges from 0 to 100, where 0 denotes no improvement and higher scores indicate improvement in pain and function [19].

SPADI Score

SPADI is a 13-item patient-reported outcome (PRO) instrument specifically designed and developed to measure pain level and extent of difficulty with ADLs. The pain subscale has five items and the disability subscale has eight items. The overall SPADI score ranges from 0 to 130, where 0 denotes less shoulder disability and higher scores indicate more shoulder dysfunction [20].

SST Score

SST is a shoulder-specific PRO tool that employs 12 questions on pain and function of the shoulder. It is a short and simple survey that requires answers as yes or no [21].

SANE Score

SANE is a simple method of evaluating patients’ sense of functional improvement after rotator cuff repair surgery, ranging from 0 (no improvement) to 100 (good improvement with normal functioning) [22].

Statistical analysis

Statistical analysis was performed using GraphPad software v8.0. Descriptive data were collected and reported as mean ± standard deviation, range, proportions, and percentages.

## Results

Disposition and demographics

A total of 39 patients who underwent rotator cuff repair surgery with Ceptre® Titanium Screw Anchor between January 2019 and July 2022 were enrolled in the study. All patients were followed up telephonically in December 2022, and no patient was lost to follow-up.

For baseline parameters, the mean age of the recruited patients was 59.74 ± 8.91 years (range = 27-73 years), with 64% of female preponderance (n = 25/39). The remaining 14 (36%) patients were male. About 90% (n = 35) of patients were aged 50 years or above. All 39 (100%) participants were married. Regarding occupation, only four (10%) patients were businessmen or working employees, and the other 35 (90%) patients were non-workers. Among the non-working patients, 24 (61%) were housewives, and 11 (28%) patients had retired from their work. The majority of the patients (87%; n = 34/39) were from Gujarat, three patients were from Rajasthan, and two patients were from West Bengal. The demographic characteristics of the patients are presented in Table 1.

**Table 1:** Demographic characteristics of the patients. n = number of patients; % = percentage; SD = standard deviation

Characteristics, n (%)	Number of patients (n = 39)
Age (years; mean ± SD)	59.74 ± 8.91
<50 years	04 (10)
50 years	35 (90)
Gender	
Male	14 (36)
Female	25 (64)
Ethnicity: Asian	39 (100)
Marital status: Married	39 (100)
Employment status	
Full working (>8 hours)	24 (46)
Not working	28 (54)
Occupation	
Business	03 (08)
Doctor	01 (03)
Household	24 (61)
Retired employees	11 (28)
Location/Residence	
Gujarat	34 (87)
Rajasthan	03 (08)
West Bengal	02 (05)

Clinical representation

About 85% (n = 33) of patients had a right shoulder injury, whereas 15% (n = 6/39) of patients had a left shoulder injury. In all patients, the right shoulder was dominant and the left part was non-dominant. The majority of patients had a complete tear (n = 38/39), and only one patient had an incomplete type of tear. All patients represented symptomatic effects. Further, the mean time period of injury to the date of surgery was 4.44 ± 2.55 months (Table 2).

**Table 2:** Clinical representation of the patients. n = number of patients; % = percentage; SD = standard deviation

Characteristics, n (%)	Number of patients (n= 39)
Shoulder injury	
Right	33 (85)
Left	06 (15)
Affected dominant side	
Dominant	33 (85)
Non-dominant	06 (15)
Type of tear	
Complete	38 (97)
Incomplete	01 (3)
Time of surgery since injury (months; mean ± SD)	4.44 ± 2.55

RCT surgery details

All patients underwent MRI to diagnose the type of RCT such as supraspinatus tear, supraspinatus + infraspinatus tear, subscapularis tear, biceps tendon partial tear, and long hand biceps tendon tear. About 64% (n = 25/39) of patients had supraspinatus tears, whereas 36% (n = 14) of patients had both supraspinatus and infraspinatus tears. All patients underwent associated surgeries including biceps tenotomy, subacromial deconstruction, and subscapularis repair. The mean mediolateral retraction tear size was 1.08 ± 0.56 and the mean anteroposterior width tear size was 2.03 ± 0.60. The mean number of days hospitalized was 2.05 ± 0.22 days (Table 3).

**Table 3:** RCT surgery details. MRI = magnetic resonance imaging; RCT = rotator cuff tears; cm = centimeter; n = number of patients; % = percentage; SD = standard deviation

Characteristics, n (%)	Number of patients (n= 39)
MRI findings	
Supraspinatus tear	25 (64)
Supraspinatus + infraspinatus tear	12 (31)
Subscapularis + supraspinatus + infraspinatus tear	02 (5)
Rotator cuff tendon involved	
Supraspinatus	25 (64)
Supraspinatus + infraspinatus	14 (36)
Length of tear (cm, mean ± SD)	
Mediolateral retraction	1.08 ± 0.56
Anteroposterior width	2.03 ± 0.60
Number of days hospitalized (days, mean ± SD)	2.05 ± 0.22
Associated surgeries	
Biceps tenotomy + subacromial deconstruction	37 (95)
Subscapularis repair + biceps tenotomy + subacromial deconstruction	02 (5)
Number of implants	
2	37 (95)
3	02 (5)

Patient-reported instruments and outcomes

The different score values as functional outcomes of rotator cuff repair surgery are shown in Table 4.

**Table 4:** Functional outcomes of rotator cuff surgery. ASES = American Shoulder and Elbow Surgeons; SPADI = Shoulder Pain and Disability Index; SST = Simple Shoulder Test; SANE = Single Assessment Numeric Evaluation; n = number of patients; % = percentage; SD = standard deviation

Parameters (mean ± SD)	Number of patients (n = 39)
Pain level, n (%)	
No	10 (26)
Mild	27 (69)
Moderate	02 (5)
ASES score	81.72 ± 14.23
SPADI score	29.41 ± 12.60
Pain score	37.75 ± 16.22
Disability score	24.04 ± 12.30
SST score	75.41 ± 12.96
SANE score	94.67 ± 7.50

ASES Score

The subjective shoulder function was assessed postoperatively using the ASES score. The mean ASES score was 81.43 ± 14.20. About 28% (n = 11) of patients had ASES scores ranging above 90, 44% (n = 17) had scores of 80-89, and 28% (n = 11) of patients had scores below 80.

Shoulder Pain Level

During the telephonic follow-up, 10 (out of 39; 26%) patients reported no pain, 27 (69%) patients reported mild pain, and the remaining two (5%) patients reported moderate pain.

SPADI Score

**Figure 2:**
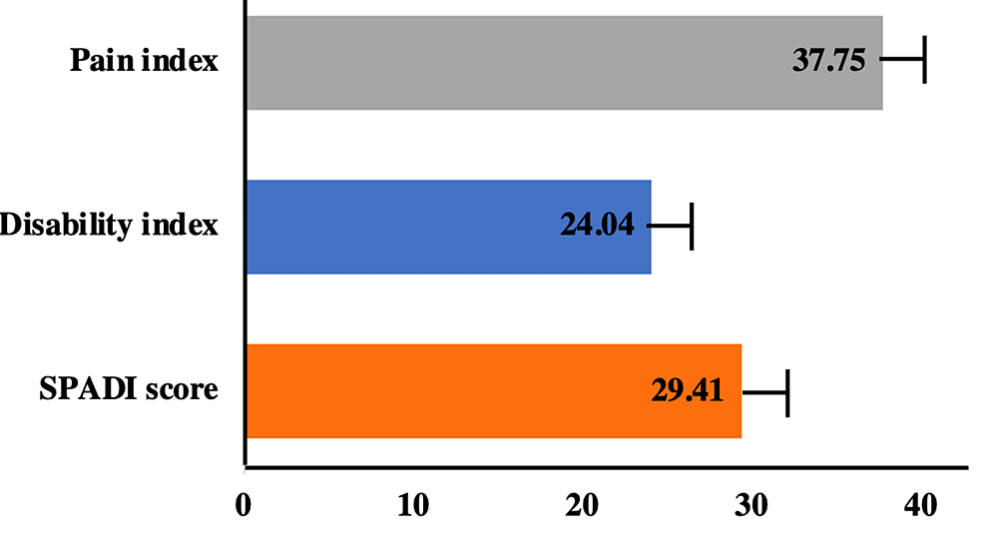
Functional outcomes of rotator cuff surgery using the SPADI score. The bar graph represents the percentage of patients showing functional outcomes of rotator cuff surgery using the SPADI score. SPADI = Shoulder Pain and Disability Index

SPADI score consists of two indices, i.e., pain and disability. The postoperative mean SPADI score of the patients was found to be 29.41 ± 12.6. When the different indices were recapitulated, the mean pain score was found to be 37.75 ± 16.22, and the mean disability score was 24.04 ± 12.30 (Figure 2).

SST and SANE Score

The mean scores of SST and SANE were calculated as 75.41 ± 12.96 and 94.67 ± 7.50, respectively.

Adverse Events

Patients were followed up for a mean duration of 20.56 ± 10.69 months. Lastly, no adverse events, re-tears, re-injuries, or re-surgeries were reported by any of the patients after surgery.

## Discussion

RCTs are a common source of shoulder pain. The incidence of rotator cuff damage increases with age and is most frequently due to degeneration of the tendon [23]. RCTs can be treated arthroscopically using different types of implants. In this study, Ceptre titanium screw anchors were used, and functional outcomes were assessed using ASES, SPADI, SST, and SANE scores.

A total of 39 patients who underwent rotator cuff repair surgery between January 2019 and July 2022 were enrolled in the study. All recruited patients were aged between 27 and 73 years. At the time of surgery, the mean age of the recruited patients was 59.74 ± 8.91 years. Data in the literature suggest that the incidence of RCT increases with age and is more commonly found in those aged 50 years and above [23]. We also observed that 90% of the recruited patients in our study belonged to this age group. Similar to our findings, a study by Vikram and Anshuman (2020) recruited 40 patients with a mean age of 59 years who underwent rotator cuff repair surgery with a single or double-row suture anchor technique [24]. Among the recruited patients, 64% were females and 36% were males. All recruited participants were Indian.

In our study, 85% of patients had right shoulder injuries, whereas the remaining 15% of patients (n = 6/39) had left shoulder injuries. Further, the mean time period of injury to the date of surgery was 4.44 ± 2.55 months. When the ideal time between the injury and surgery was estimated, there was no clear-cut consensus on this [25,26].

Further, 64% (n = 25/39) of patients had supraspinatus tears, whereas 36% (n = 14) of patients had both supraspinatus and infraspinatus tears. The study by Vikram and Anshuman observed that 62.5% of patients had only supraspinatus tears and 37.5% of patients had both supra and infraspinatus tears among the 40 recruited patients. The data is in accordance with our study [24].

Thereafter, the ASES score was used to assess the functional outcomes of the rotator cuff surgery. The mean ASES score was 81.43 ±14.20. In a study, Assunção et al. (2017) recruited 143 patients and evaluated the clinical outcomes of arthroscopic rotator cuff repair using the University of California at Los Angeles (UCLA) and ASES scores. The authors reported an ASES score of 81.2 ± 20.8 after 24 months of surgery [27]. The data is in concordance with our findings. In another study, Kim et al. (2019) evaluated the ASES score in 30 patients who underwent RCT using arthroscopic suture-bridge repair. The authors found an ASES score of 72.8 ± 20.5, which is less compared to our findings [28].

During the telephonic follow-up, 10 (out of 39; 26%) patients reported no pain, 27 (69%) patients reported mild pain, and the remaining two (5%) patients reported moderate pain. Further, the SPADI score was calculated. SPADI score consists of two indices, i.e., pain and disability. The postoperative mean SPADI score of the patients was 29.41 with a standard deviation of 12.6. When the different indices were recapitulated, the mean pain score was 37.75 ±16.22 and the mean disability score was 24.04 ± 12.30. In a study, Hopewell et al. (2021) perform the GRASP (Getting it Right: Addressing Shoulder Pain) study for the treatment of RCT. The authors observed a mean SPADI score of 30 in their recruited patients after treatment during follow-up at 12 months and above. The scores of the GRASP study are similar to our data [29]. 

A systematic review of 654 studies using the SST demonstrated that this outcome score is commonly used to evaluate patients with rotator cuff disease (174 studies [27%]) and those with arthritis and arthroplasty (153 studies [23%]) [21]. Further, in our study, the mean scores of SST and SANE parameters were 75.41 ± 12.96 and 94.67 ± 7.50, respectively. In a study, Mijic et al. (2020) investigated the effect of biceps tenodesis on the speed of recovery after arthroscopic rotator cuff repair. The authors observed a mean SST score of 75 and 83 during follow-ups at six months and 12 months, respectively, which is similar to our data. The mean SANE score was found to be 80 and 84 at six and 12 months of follow-up in the study of Mijic et al. (2020) [30]. However, the data in our study was higher compared to this study.

Lastly, no adverse events, postoperative complications, re-injuries, or re-surgeries were reported by any of the patients during the follow-up. Similarly, Vikram and Anshuman reported no postoperative complications in their study [24]. However, in two different studies by Park et al. (2015) and Kim et al. (2018), who used single-row anchor suture and trans-tendon suture bridge for RCT repair in 33 patients, re-tear rates of 18.5% and 6.1%, respectively, were reported [28,31].

Limitations

There are a few limitations of this study. The study had a small sample size and the data were collected through telephonic follow-up. Therefore, no physical verification of the data could be done. As the study was retrospective, no control group could be recruited to compare the safety, functionality, and success of the Ceptre implant.

## Conclusions

Arthroscopic rotator cuff repair surgery resulted in favorable functional outcomes with good patient-outcome scorings. The results of this study suggest that arthroscopic rotator cuff repair using Ceptre Knotted UHMWPE Suture Titanium Screw Anchor may be a considerable option for a successful surgery. Further studies are needed to assess the long-term stability, safety, and efficacy of the Ceptre implant in rotator cuff repair surgeries.
